# Do frontline health care providers know enough about artemisinin–based combination therapy to rationally treat malaria? A cross-sectional survey in Gezira State, Sudan

**DOI:** 10.1186/s12936-015-0652-0

**Published:** 2015-03-26

**Authors:** Abeer A Mannan, Khalid A Elmardi, Yassir A Idris, Jonathan M Spector, Nahid A Ali, Elfatih M Malik

**Affiliations:** Al Neelain University, Steen Street, P.O. Box 7294, Code: 11123 Khartoum, Sudan; Federal Ministry of Health, Khartoum, Sudan; Gezira State Ministry of Health, Gezira, Sudan; Massachusetts General Hospital, 55 Fruit Street, Boston, MA 02114 USA

## Abstract

**Background:**

In 2004, artemisinin-based combination therapy (ACT) was introduced in Sudan for the treatment of malaria. The role of health care providers working in first-level health care facilities is central for the effective implementation of this revised malaria treatment policy. However, information about their level of ACT knowledge is inadequate. This study sought to describe frontline health care providers’ knowledge about the formulations and dose regimens of nationally recommended ACT in Sudan.

**Methods:**

This cross-sectional study took place in Gezira State, Sudan. Data were gathered from five localities comprising forty primary health care facilities. A total of 119 health care providers participated in the study (72 prescribers and 47 dispensers). The primary outcome was the proportion of health care providers who were ACT knowledgeable, a composite indicator of health care providers’ ability to (1) define what combination therapy is; (2) identify the recommended first- and second-line treatments; and (3) correctly state the dose regimens for each.

**Results:**

All prescribers and 95.7% (46/47) of dispensers were aware of the new national malaria treatment policy. However, 93.1% (67/72) of prescribers compared to 87.2% (41/47) of dispensers recognized artesunate-sulphadoxine/pyrimethamine as the recommended first-line treatment in Sudan. Only a small number of prescribers and dispensers (9.4% and 13.6%, respectively) were able to correctly define the meaning of a combination therapy. Overall, only 22% (26/119, 95% CI 14.6-29.4) of health care providers were found to be ACT knowledgeable with no statistically significant difference between prescribers and dispensers.

**Conclusion:**

Overall, ACT knowledge among frontline health care providers is very poor. This finding suggests that efforts are needed to improve knowledge of prescribers and dispensers working in first-level health care facilities, perhaps through implementing focused, provider-oriented training programmes. Additionally, a system for regularly monitoring and evaluating the quality of in-service training may be beneficial to ensure its responsiveness to the needs of the target health care providers.

## Background

Malaria continues to be a major public health problem in most African countries including Sudan [1,2]. In 2001, the WHO recommended the use of artemisinin–based combination therapy (ACT) for treating malaria due to its rapid clearance of parasitaemia and high cure rate [3-5]. While costly compared with other anti-malarials, ACT is highly efficacious and safe [6-8]. In 2004, Sudan changed its malaria treatment policy and switched to ACT [9]. Currently, the nationally recommended first line and second line treatments are artesunate-sulphadoxine/pyrimethamine (ASP) and artemether-lumefantrine (AL), respectively. ASP is widely accessible and offered free of charge in primary health care facilities in Sudan. However, the successful implementation of the ACT treatment policy largely depends upon adequate training and support for health care providers to improve their knowledge and to ensure their acceptance and adherence to the new treatment guidelines [10]. Providers’ correct knowledge of treatment guidelines has been cited in the literature as an important prerequisite for effective malaria case management [11]. Nationally, no sufficient information is available that describes how much prescribers and dispensers of ACT know about this effective treatment, which is currently the best hope for treating malaria worldwide. This study was undertaken to assess the level of knowledge of health care providers working in public primary health care units about ACT in terms of its definition, recommended formulations, and dosages. Primary health care providers were specifically targeted given their role in the workforce on the frontlines where they manage the greatest proportion of malaria patients. Primary health care providers have a responsibility to the welfare of their patients through regularly updating their knowledge to ensure proper malaria case management. Although correct knowledge of health care providers does not necessarily mean adherence to treatment guidelines [12,13], determining their level of knowledge is essential for understanding the malaria treatment situation in Sudan. It was anticipated that the results of this survey would contribute to the evidence describing the use of ACT in Sudan, and would inform the need for possible investment in targeted training programmes to ensure the delivery of better quality malaria treatment services.

## Methods

### Study setting

This survey was carried out in Gezira state, Sudan. Data were collected from 40 primary health care facilities located in five localities; namely Greater Medani, Gezira south, Gezira east, Hasheesa, and Kamleen .

### Study population

A multi-stage sampling technique was employed to select the study participants. In the first stage, five localities were randomly selected. In the second stage, a sample of 40 primary health care facilities was randomly drawn. In the final stage, all health care providers (prescribers and dispensers) working in the selected health facilities at the time of the study were interviewed.

### Data collection

A structured interview was conducted to assess three dimensions of health care providers’ knowledge using pre-coded and pre-tested questionnaire. First, their general knowledge regarding ACT definition and its introduction as the first-line treatment in Sudan was assessed. Second, knowledge about the routes and dosages of the nationally recommended first-, second- and third-line treatments was assessed. Third, it was investigated whether they perceived the ACT treatment guidelines as effective, and the ACT wall charts as clear and informative. For the prescribers, an additional dimension was explored, which was their background knowledge about malaria case definition, mode of diagnosis, and their prescribing habits.

### Data analysis

The main outcome of this study was the proportion of health care providers who demonstrated an adequate knowledge about ACT guidelines for treating uncomplicated malaria. This was defined by health care providers’ ability to (1) define what combination therapy is; (2) identify the recommended first- and second-line treatments; and (3) correctly state the dose regimens for each. Data were entered and analysed using SPSS version 22.

### Ethical approval

Ethical approval for this study was provided by the Al Neelain University institutional review board, the National Fund for Promoting Medical Services, and the Gezira State Ministry of Health. The purpose of the study was fully explained to all participants, and their verbal consent was obtained prior to conducting the interviews.

## Results

A total of 72 prescribers and 47 dispensers participated in the study. All prescribers and 95.7% (46/47) of dispensers were aware of the national malaria treatment guidelines (Table 1). ASP was identified as the recommended first-line treatment in Sudan by 93.1% (67/72) of prescribers and 87.2% (41/47) of dispensers. With the exception of only one prescriber, all health care providers indicated the oral route as the recommended mode of ASP administration. Over 30% (83/119) of health care providers were unable to distinguish the difference between ASP as a combination therapy and the formerly recommended first-line anti-malarial (i.e. chloroquine). A very few number of prescribers and dispensers (9.4% and 13.6%, respectively) were able to correctly define the meaning of a combination therapy.

**Table 1:** **Knowledge of health care providers (HPs) about ACT treatment policy**

**Domain**	**Prescribers**	**Dispensers**	**Total**	**95% CI**	**Chi-square**	**P. value**
	**N = 72**	**N = 47**	**N = 119**			
	**n (%)**	**n (%)**	**n (%)**			
HP heard of new national treatment guidelines for malaria	72	46	118	97.6-100	1.545	0.395
	100	95.7	99.2			
HP recognized Artesunate- sulphadoxine/pyrimethamine (ASP) as the recommended first-line treatment for malaria	67	41	108	85.6-96	3.495	0.321
	93.1	87.2	90.8			
HP identified ASP as a combination therapy	51	32	83	61.4-78	1.667	0.644
	70.8	68.1	69.7			
HP defined a Combination therapy correctly	7	6	13	5.3-16.5	2.727	0.604
	9.4	13.6	10.9			
HP recognized the oral route for ASP as the nationally recommended	71	47	118	97.6-100	0.658	1
	98.6	100	99.2			

Although the majority of heath care providers recognized ASP as the first-line treatment, their knowledge about the recommended dosage in milligrams and the number of tablets in a full course was very low in both groups (Table 2).

**Table 2:** **Knowledge of health care providers (HPs) about the routes and doses of the; first, second and third line treatments**

**Domain**	**Prescribers**	**Dispensers**	**Total**	**95% CI**	**Chi-square**	**P. value**
	**N = 72**	**N = 47**	**N = 119**			
	**n (%)**	**n (%)**	**n (%)**			
HP recognized Artesunate-sulphadoxine/pyrimethamine (ASP) as the first-line treatment	67	43	110	87.6-97.2	0.332	0.847
	93.1	91.5	92.4			
HP stated the recommended dose for ASP in mg correctly	22	13	35	21.9-38	1.787	0.618
	32.8	29.5	29.7			
HP recognized how ASP is taken correctly	44	24	68	49.0-66.8	1.172	0.279
	61.1	51.1	57.1			
HP recognized Artemether + lumefantrine (AL) as the second-line treatment	48	24	72	51.7-69.3	7.084	0.069
	66.7	51.1	60.5			
HP stated the recommended dose for AL in mg correctly	26	16	42	26.7-43.9	1.800	0.407
	36.1	34	35.3			
HP recognized how AL is taken correctly.	46	24	70	50.0-67.6	1.932	0.381
	63.9	51.1	58.8			
HP recognized quinine as the third-line treatment	60	42	102	79.4-92.2	4.201	0.122
	83.3	89.4	85.7			
HP stated the recommended dose for oral quinine correctly	56	18	74	53.5-71.0	20.732	0.000
	77.8	38.3	62.2			
HP recognized the main indication for administering injectable quinine in malaria patients	43	32	75	54.3-71.1	1.358	0.715
	59.7	68.1	63			
ACT knowledgable*	16	10	26	14.6-29.4	0.026	1
	22.5	21.3	22			

*ACT knowledgeable = HP identified ACT as a combination therapy + HP recognized Artesunate-sulphadoxine/pyrimethamine (ASP) as the first-line treatment + HP recognized how ASP is taken correctly + HP recognized Artemether/lumefantrine (AL) as the second-line treatment + HP recognized how AL is taken correctly.

Regarding recommended second-line treatment, only 66.7% (48/72) of prescribers and51.1% (24/47) of dispensers were able to recognize artemether/lumefantrine (AL) as the treatment of choice. Similarly, the level of knowledge about the correct dosage and amount was very low among both prescribers and dispensers (Table 2). Overall, only 22% (26/119, 95% CI 14.6-29.4) of health care providers were considered ACT-knowledgeable with no statistically significant difference between the two groups. More prescribers participated in this study compared to dispensers. That was a true reflection of the pattern of the distribution of healthcare personnel in primary health care facilities in Sudan.

Knowledge about third-line treatment was comparably higher in both groups. Quinine tablets were identified to be the recommended third-line treatment in Sudan by83.3% (60/72) of prescribers and 89.4% (42/47) of dispensers. Regarding the recommended quinine dosage, the level of knowledge was significantly higher among prescribers (P value = 0.00). A considerably low number of health care providers (64.8% of prescribers and 68.1% of dispensers, respectively) correctly stated the indication for administering quinine injections for malaria confirmed patients.

The vast majority of prescribers (90.3%, 65/72) and dispensers (95.7%, 45/47) perceived ASP as effective. Nevertheless, only a few of them have been trained on the use of the ACT guidelines (Table 3). More dispensers (93.3%, 42/47) perceived the malaria treatment wall charts as informative and clear compared to prescribers (86.1%, 62/72), though the difference was not statistically different.

**Table 3:** **Perception of health care providers (HPs) about ASP effectiveness, malaria wall charts & training**

**Domain**	**Prescribers**	**Dispensers**	**Total**	**95% CI**	**Chi-square**	**P. value**
	**N = 72**	**N = 47**	**N = 119**			
	**n (%)**	**n (%)**	**n (%)**			
HP describes ASP as effective	65	45	110	87.6-97.2	1.216	0.480
	90.3	95.7	92.4			
HP describes the treatment guidelines wall chart as clear	62	42	104	83.3-94.5	5.931	0.204
	86.1	93.3	88.9			
HP received training on national treatment guidelines	18	9	27	15.4-30.5	0.470	0.654
	25.0	19.1	22.9			

This investigation also included additional questions specifically developed to obtain further information about prescribers’ background knowledge of malaria case definition, their attitudes towards malaria laboratory diagnosis, and whether they prescribe ASP as the first-line in their practice (Table 4). 44.4% (32/72) of prescribers adequately defined a case of malaria. Alarmingly, only 22.4% (15/72) of prescribers indicated that they rely on the laboratory results to confirm malaria diagnosis. Also, it was found that although 90.3% (65/72) of prescribers described ASP as effective, only 63.2% (47/72) of them have stated that they actually prescribe it as the first treatment option for uncomplicated malaria in their routine practice.

**Table 4:** **Prescribing behaviour of prescribers**

**Domain**	**Count**	**%**	**95% CI**
Prescriber defined a case of malaria correctly	32	44.4	32.9-55.9
Prescriber relies on laboratory results when treating malaria	15	22.4	12.8-32.0
Prescriber always prescribe ASP as the first line for uncomplicated malaria	47	63.2	52.1-74.3

## Discussion

Health care providers working in primary health care facilities in Sudan play an essential role in delivering care for patients with malaria. However, in low income-countries health care providers have been found to perform below their potential due to factors such as heavy workload coupled with shortage of staff [14,15]. Furthermore, evidence has shown that primary health care providers in resource-restricted countries sometimes lack support and can be dissatisfied [16,17]. One of the main challenges facing primary health care providers in Sudan is the rising number of malaria patients presenting to health care facilities, in particular during the rainy season [18]. Malaria treatment guidelines have changed following the introduction of ACT in 2004. This switch to a new treatment policy has been accompanied by several measures to ensure its effective implementation, including in-service training of health care providers to regularly update their knowledge and improve their skills.

The results of this study have shown a significant knowledge gap among primary health care providers about the ACT policy. Although the vast majority of prescribers and dispensers recognized the new treatment guidelines, and were able to identify oral ASP as the first-line treatment for uncomplicated malaria, a fewer number of them recognized ASP as a combination therapy. Failure to acknowledge ACT as a combination therapy is troublesome. One component of the combination therapy, S/P, was previously used as a second-line treatment. Prior knowledge about S/P might have caused some confusion for some health care providers. It is of utmost importance for health care providers to understand why the use chloroquine has been discontinued, and it is essential for them to appreciate in what ways ACT is superior to chloroquine. Health care providers are instrumental for the successful implementation of the ACT policy in Sudan and their acceptance and adherence to this policy is fundamental to realizing acceptable treatment outcomes. Prior evidence has correlated poor providers’ adherence to treatment guidelines with their inadequate knowledge and misperception [19]. Key messages related to what a combination therapy is, why it is needed, and the particular advantages it has over chloroquine should always be clearly and adequately incorporated in in-service training progammes.

Further analysis of health care providers’ responses revealed a substantial knowledge deficiency regarding the dosing regimens of the recommended first and second line treatments. Similar patterns of providers’ poor knowledge about doses, predominantly in the private sector, have been found in low-income countries in Africa [20-22]. Rigorous knowledge about treatment prescribed to patients is equally essential to both prescribers and dispensers. ASP and AL are available pre-packed and are provided free of charge in primary health facilities. Apparently, health care providers are less motivated to remember the dosages in milligrams for tablets contained inside a pre-packed envelope. In some health facilities, prescribing and dispensing of anti-malarials are both undertaken by the same health care provider, and that practice in particular has made matters worse since no prescription is needed for dispensing the anti-malarial. Dispensing anti-malarials is thus a habitual process rather than an organized professional practice in these health facilities. Haphazard use of ACT resulting from inadequate knowledge could have serious implications for patient safety as well as for emergence of parasite resistance. Training programmes may need to be more focused, and tailored according to the perceived needs of health care providers, not exclusively delivered as a basic package determined by health authorities. A training programme that is more responsive to the felt needs of the target audience is more likely to produce a positive impact and attain its goals [23]. However, to ensure safe and effective use of ACT, training alone is insufficient. Ongoing follow-up and supervision may be as important as the training itself, and in settings such as Sudan may actually be more challenging to put into practice than the initial training workshops. A clear monitoring system augmented by strict regulations is also needed to oversee practices, determine deficiencies and provide timely solutions.

Interestingly, the level of knowledge about the dosage of the third line anti-malarial (i.e. quinine) was higher among prescribers compared to their knowledge about dosages of the first- and second-line treatments. Also, it was observed that there is a statistically significant difference in quinine knowledge between prescribers and dispensers (P = 0.000). The choice of quinine as the third line treatment has not changed following the introduction of ACT in Sudan. Most prescribers are fairly familiar with this particular anti-malarial, and that could explain their improved knowledge about it. Furthermore, ACT is a relatively new drug due to its recent marketing and implementation. ACT contains more than one drug, each with a different dose schedule making it more unlikely for a health care provider to efficiently recall dosages [20,24]. Regarding the use of injectable quinine, it has been found that a large number of prescribers and dispensers have indicated unnecessary reasons for prescribing injections. Other studies have also shown that injections are often unnecessarily used [25]. Greater caution has to be taken when prescribing injectable anti-malarials to minimize the hazards associated with this type of preparations. This information should be explicitly and strictly conveyed to health care providers.

Overall, the primary outcome of this study was the proportion of health care providers who were ACT knowledgeable. Considerably few health care providers demonstrated adequate knowledge about ACT and could, therefore, be labelled ‘ACT–knowledgeable’. This is consistent with prior evidence of substantially low level of knowledge about malaria treatment guidelines in other countries [26]. The finding sounds alarm bells and may suggest the need for more effective training and supervision to enhance health care providers’ knowledge and practice.

Regarding health care providers perceptions of the ACT guidelines, it was found that the majority of both prescribers and dispensers perceive ASP as effective. The majority described the ACT guidelines wall chart as clear and informative. Wall charts were present in all study health facilities at the time data were collected. Wall charts are helpful aids that can be used by prescribers and dispensers to readily recall key information. However, while health care providers have a positive attitude towards wall charts they do not seem to have effectively assimilated the information contained in these charts. Inadequate training is possibly the main hurdle as most of prescribers and dispensers indicated that they have not received training on the ACT guidelines. A previous study in Malawi has shown similar results [27]. In-service training is one of the key activities delivered by Gezira state malaria control programme on an ongoing basis, though adequate information about the impact that training has on the performance of health care providers is lacking. A systematic training needs assessment should be undertaken prior to conducting in-service training to ensure its effectiveness.

In addition to examining prescribers’knowledge about ACT, this study explored other facets related to their prescribing behaviour; specifically, under what conditions malaria should be suspected, how it should be diagnosed, and the willingness with which the recommended first-line is prescribed. Surprisingly, the majority of prescribers failed to define a case of malaria correctly. Moreover, most prescribers lacked confidence in laboratory diagnosis and did not often rely on such results for confirming a diagnosis of malaria. Similar patterns have been observed in Nigeria [28]. More strict regulations are needed to control prescribing behaviour of health care providers, particularly at primary level were most malaria patients are seen. Parasitological testing of malaria has been found to effectively reduce the number of anti-malarial prescriptions [29] and it continues to be the gold standard for confirming the diagnosis of malaria. However, it was extremely worrying to find that although over 90% of prescribers perceived ASP as effective only 63.2% actually prescribe it as the first choice. It is, therefore, worth mentioning that a strong positive perception about a treatment does not necessarily translate into adherence to its guidelines. Several studies have indicated that changing the behaviour of health care providers is challenging [17,30]. More in-depth analysis is needed to find out why some prescribers are so reluctant to prescribe ASP in order to avoid repeating the failures of the past related to non-compliance to nationally recommended treatment guidelines.

## Conclusions

The overall level of knowledge about ACT among prescribers and dispensers is critically low. Investment in focused training and regular supervision may be needed, particularly for first-level health care providers on the frontlines who care for the greatest proportion of malaria patients. In addition, further research is needed to identify how best to translate improved providers’ knowledge into better practice. The goal is substantial reduction of malaria burden on the national health system in Sudan.
